# Role of corneal radius of curvature in early identification of fundus tessellation in children with low myopia

**DOI:** 10.1136/bjo-2022-321295

**Published:** 2022-07-26

**Authors:** Wei Gong, Tianyu Cheng, Jingjing Wang, Bo Zhang, Jun Chen, Jianfeng Zhu, Haidong Zou, Kun Liu, Xiangui He, Xun Xu

**Affiliations:** 1 Department of Ophthalmology, Shanghai General Hospital, Shanghai Jiao Tong University School of Medicine, National Clinical Research Center for Eye Diseases, Center of Eye Shanghai Key Laboratory of Ocular Fundus Diseases, Shanghai Engineering Center for Visual Science and Photomedicine, Shanghai, People's Republic of China; 2 Department of Clinical Research, Shanghai Eye Disease Prevention and Treatment Center, Shanghai Eye Hospital, Shanghai Vision Health Center & Shanghai Children Myopia Institute, Shanghai, People's Republic of China

**Keywords:** Choroid, Diagnostic tests/Investigation, Retina, Child health (paediatrics), Epidemiology

## Abstract

**Aim:**

To assess the role of the corneal radius of curvature (CR) in the identification of fundus tessellation in children with low myopia.

**Methods:**

In the cross-sectional study, students aged 9–12 years from 24 primary schools in Shanghai were enrolled by cluster sampling. Participants underwent measurements including cycloplegic refraction and axial length. Fundus images and choroidal thickness were obtained by swept-source optical coherence tomography. Fundus tessellation was classified into four grades according to fundus photographs.

**Results:**

A total of 1127 children with low myopia (spherical equivalence (SE) >−3.00 dioptre (D) but ≤−0.50 D) were included, with a mean age of 10.29±0.60 years and a mean SE of −1.44±0.69 D. Fundus tessellation was found in 591 (52.4%) cases (grade 1: 428, 38.0%; grade 2: 128, 11.4%; grade 3: 35, 3.1%). Choroidal thickness decreased as fundus tessellation grade increased (p trend <0.001). According to regression analysis, higher fundus tessellation grade was independently associated with larger CR (OR, 7.499; 95% CI 2.279 to 24.675, p=0.001). For those with CR >7.9 mm, along with CR, degree and proportion of fundus tessellation increased sharply.

**Conclusion:**

Fundus tessellation existed in more than half of children with low myopia. Preliminary fundus photography conducted in children with low myopia with large CR would be necessary and beneficial to the early management of myopic fundus changes.

**Trial registration number** NCT02980445.

**WHAT IS ALREADY KNOWN ON THIS TOPIC**

- Fundus tessellation is an early fundus manifestation of myopia which could play a warning role in myopic maculopathy.

**WHAT THIS STUDY ADDS**

- This study specifically investigated the prevalence of fundus tessellation in children with low myopia, and there was a tendency towards maculopathy in children with low myopia with large corneal radius of curvature.

**HOW THIS STUDY MIGHT AFFECT RESEARCH, PRACTICE OR POLICY**

- Corneal radius of curvature could be advocated as a more convenient indicator for fundus photography, and a corneal radius of curvature greater than 7.9 mm in children with low myopia could be used to identify fundus tessellation.

## Introduction

Myopia is one of the major public health problems worldwide.^1 2^ In recent years, the prevalence of myopia has increased sharply and the onset age is becoming younger.^3^ A previous study predicted that without effective intervention half of the population in the world would suffer from myopia by 2050.^4^ Especially in Southeast Asia and East Asia, 10%-40% of children has myopia, and the prevalence of myopia in high school graduates reached more than 95%, among which 10%–20% were high myopia.^5–11^

Myopic maculopathy, a fundus complication of myopia, could cause irreversible visual impairment and bring heavy burden to both patients and society.^12^ Fundus tessellation is the early phase of myopic maculopathy, which refers to the choroidal vessels that can be observed on fundus photography, and could play a warning role in further myopic fundus changes.^13–15^

Fundus tessellation has been well investigated among children and adults with high myopia in previous studies and is mainly considered the consequence of axial length (AL) elongation during myopia development.^15–18^ However, in one of our paediatric cohorts (aged 9–12 years), we found that fundus tessellation commonly existed in those with low myopia (spherical equivalence (SE) >−3.00 dioptre (D) but ≤−0.50 D), which has been rarely reported to date. In the preliminary observation of the current cohort, patients with low myopia with fundus tessellation had not only longer AL but also larger corneal radius of curvature (CR) compared with those without fundus tessellation. We supposed that large CR could cover up the effect of AL elongation on myopia progression, which might result in neglect of fundus screening, especially among some children with low myopia. However, the relationship between fundus tessellation and CR among those with low myopia remains to be explored.

Therefore, this study aimed to describe the prevalence of fundus tessellation and the role of CR in the identification of fundus tessellation in children with low myopia, which might contribute to the early prevention of myopic maculopathy and visual health management.

## Methods

### Participants

In the cross-sectional study, students aged 9–12 years from 24 primary schools in Shanghai were enrolled by cluster sampling in 2019. Low myopia was defined as SE >−3.00 D but ≤−0.50 D after cycloplegia. Those with other organic eye diseases (amblyopia, strabismus, congenital cataract and glaucoma) or unqualified fundus photographs were excluded. The study protocol was explained to all participants and their guardians. Written informed consent forms were obtained from participants’ parents or legal guardians and oral consent was assented by participants. The study was registered at ClinicalTrials.gov (identifier: NCT02980445).

### Examinations

All participants underwent a series of examinations, including measurement of height and weight, intraocular pressure (Non-Contact Tonometer, NT-510, Nidek, Japan), AL (IOL Master 700, Carl Zeiss Meditec, Germany) and cycloplegic refraction.

For cycloplegia, after a slit lamp examination to guarantee the safety of cycloplegia, one drop of 0.5% proparacaine (Alcaine, Alcon) was given to each eye, followed by two drops of 1% cyclopentolate (Cyclogyl, Alcon), 5 min apart. After approximately 30 min, the absence of light reflex and a pupil diameter larger than 6 mm were considered as complete cycloplegia. Otherwise, another drop of cyclopentolate would be given. Then, refraction and corneal curvature measurements were acquired by an autorefractor (KR-8900, Topcon, Japan). Finally, a swept-source optical coherence tomography (SS-OCT; DRI OCT Triton, Topcon) examination was performed by an experienced examiner from 10:00 to 15:00 each day to minimise the influence of diurnal variation.^16^ Meanwhile, fundus photographs of the macular areas were acquired with a digital retinal camera in the same SS-OCT system.

All instruments were calibrated before examination. Three measurements were obtained from each participant, and the procedure was repeated if the difference between any two records of spherical power or cylindrical power was larger than 0.50 D, or any two records of AL or CR were larger than 0.02 mm. The mean value of the three measurements was used in the analysis.

### Choroidal thickness measurements

The SS-OCT examination was conducted in a 12-line radial scan pattern on the central fundus with a resolution of 1024 for each line. Before scanning, some ocular parameters, such as spherical power (D), cylindrical power (D), AL (mm) and CR (mm), were input to the OCT system to perform calibration for the magnification error.

Segmentation of the different layers on the OCT images was automatically completed by built-in software, followed by manual inspection and correction for misjudgement of the layer borders by an OCT technician. Images with poor quality (signal strength less than 60) were excluded from the final analysis. The ETDRS grid was centred on the fovea, separating this region into nine sectors. The average choroidal thickness (ChT) in each sector was automatically calculated by the built-in SS-OCT software.

### Grading of fundus tessellation

**Figure 1:**
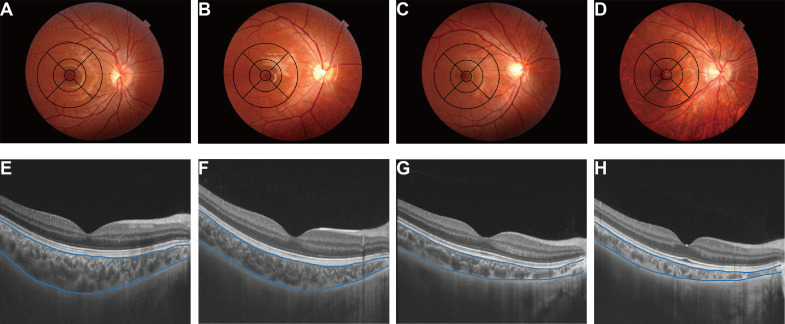
Application of ETDRS grid in fundus tessellation grading. Sample graphs and corresponding choroidal thickness for grade 0 (no involvement of the outer circle) (A and E), grade 1 (involvement of the outer circle) (B and F), grade 2 (involvement of the middle circle) (C and G) and grade 3 (involvement of the inner circle) (D and H), respectively. Average macular choroidal thickness of the OCT images: E, 319.70 µm (grade 0); F, 228.40 µm (grade 1); G, 159.80 µm (grade 2); H, 123.40 µm (grade 3). OCT, optical coherence tomography.

Fundus tessellation was graded using colour central fundus photographs obtained during the same SS-OCT examination. With an ETDRS grid (6×6 mm) centred on the fovea, fundus tessellation was classified into four grades (figure 1): no involvement of the outer circle (grade 0, including eyes without tessellation); involvement of the outer circle but not the middle circle (grade 1); involvement of the middle circle but not the inner circle (grade 2); and involvement of the inner circle (grade 3).

When assessing fundus tessellation grade, the contrast, brightness, background pigmentation and quality of the images were all taken into account. Two trained ophthalmologists (WG and TC) independently read these photographs to determine the tessellation grade. A senior ophthalmologist (XX) was required to make the decision if there was any disagreement. Intraobserver variability was tested by an ophthalmologist who randomly chose 50 of these photographs and read them twice at an interval of 2 weeks. Interobserver variability was tested by two ophthalmologists with another 50 randomly chosen photographs. The intraobserver and interobserver agreement rates for fundus tessellation grading were 0.98 (kappa=0.94) and 0.96 (kappa=0.90), respectively.

### Statistical analysis

Statistical analyses were performed with SPSS V.25.0. Only data from the right eyes were chosen and included in the final analyses. Age was calculated according to the birthday and the examination date. Continuous variables were described as mean±SD, while discrete variables were described as counts (proportions). SE was calculated as spherical power+0.5×cylindrical power. Body mass index (BMI) was calculated as weight/height^2^ (kg/m^2^). The subjects were grouped according to AL (23.0≤AL<23.5 mm; 23.5≤AL<24.0 mm; 24.0≤AL<24.5 mm; 24.5≤AL<25.0 mm; AL ≥25.0 mm) (p<0.001) and CR (CR <7.8 mm; 7.8≤CR<7.9 mm; 7.9≤CR<8.0 mm; 8.0≤CR<8.1 mm, 59.3%; 8.1≤CR<8.2 mm; CR ≥8.2 mm).

## Results

### General characteristics

**Figure 2:**
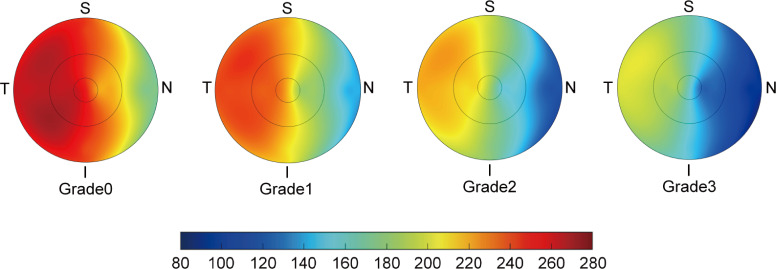
Choroidal thickness in different fundus tessellation grades. Topographic pictures of choroidal thickness in the ETDRS grid centred on the fovea, from grade 0 to grade 3. The value of choroidal thickness is shown in different colours. I, inferior; N, nasal; S, superior; T, temporal.

A total of 1155 participants with low myopia from one of our large-scale cohorts were enrolled and 28 participants who met the exclusion criterion were excluded. Finally, 1127 participants were included in the analysis. As shown in table 1, the mean age was 10.29±0.60 years (range 9.15–12.59 years) and 542 (48.1%) were boys. The mean AL was 24.17±0.74 mm; the mean SE was −1.44±0.69 D; the mean CR was 7.82±0.25 mm; and the mean macular (inside the outer circle of the ETDRS grid) ChT was 214.36±42.08 µm. Boys had larger BMI, AL, CR and AL/CR than girls, but had thinner subfoveal ChT (all p<0.001). Fundus tessellation in the macular area was found in 591 subjects (52.4%), including 428 grade 1 (38.0%), 128 grade 2 (11.4%) and 35 grade 3 (3.1%) (figure 2).

**Table 1:** Systemic and ophthalmological parameters of children with low myopia

Variables	Total (N=1127)	Boys (n=542)	Girls (n=585)	P value*
Systemic parameters				
Age, years	10.29±0.60	10.30±0.60	10.27±0.60	0.514
Height, cm	143.23±7.42	142.79±6.96	143.64±7.80	0.053
Weight, kg	38.90±9.50	39.84±9.71	38.04±9.22	**0.001**
Body mass index, kg/cm²	18.77±3.40	19.36±3.60	18.24±3.12	**<0.001**
Ophthalmological parameters				
Spherical equivalent, dioptre	−1.44±0.69	−1.44±0.69	−1.44±0.69	0.997
Axial length, mm	24.17±0.74	24.45±0.72	23.91±0.65	**<0.001**
Corneal radius of curvature, mm	7.82±0.25	7.88±0.25	7.76±0.23	**<0.001**
Axial length/corneal radius of curvature	3.09±0.07	3.10±0.07	3.08±0.07	**<0.001**
Macular choroidal thickness, μm	214.36±42.08	212.66±42.47	215.95±41.68	0.192
Macular retinal thickness, μm	276.24±11.40	277.10±11.31	275.45±11.44	**0.016**
Subfoveal choroidal thickness, μm	227.89±52.41	225.84±53.27	229.80±51.56	**<0.001**
Foveal retinal thickness, μm	231.41±16.49	233.94±16.24	229.06±16.38	0.206
Fundus tessellation, n (%)				
Grade 0	536 (47.6)	245 (49.7)	291 (45.2)	0.48
Grade 1	428 (38.0)	213 (36.8)	215 (39.3)	
Grade 2	128 (11.4)	66 (10.6)	62 (12.2)	
Grade 3	35 (3.1)	18 (2.9)	17 (3.3)	

P values < 0.05 were in bold.

*T-test or χ^2^ test to compare systemic or ophthalmological parameters between boys and girls.

### Comparison of parameters by different fundus tessellation grades

There were significant differences in AL, CR, macular ChT, macular retinal thickness and subfoveal ChT among the different fundus tessellation grades (all p<0.001, p trend <0.001), but none for age, sex, height, weight, BMI, SE, AL/CR and foveal retinal thickness (table 2, figure 2). The mean ChT decreased from 234.07±40.13 µm in grade 0 to 205.04±33.24 µm in grade 1, to 179.28±29.95 µm in grade 2 and to 154.15±22.70 µm in grade 3 (p<0.001). There was also a downward trend in the mean values of subfoveal ChT and macular retinal thickness with higher fundus tessellation grades (all p<0.001).

**Table 2:** Systemic and ophthalmological parameters among different fundus tessellation grades

Variables	Fundus tessellation grade				P value*	P trend†	Post-hoc test‡
	Grade 0	Grade 1	Grade 2	Grade 3			
Systemic parameters							
Age, years	10.26±0.62	10.31±0.56	10.32±0.65	10.33±0.49	0.399	0.451	–
Boys, n (%)	245 (45.7)	213 (49.8)	66 (51.6)	18 (51.4)	0.480	0.140	–
Height, cm	143.43±7.29	143.16±7.38	142.50±7.40	143.67±9.66	0.619	0.992	–
Weight, kg	39.19±9.34	38.86±9.66	37.65±8.49	39.74±12.84	0.392	0.928	–
Body mass index, kg/cm²	18.88±3.41	18.77±3.45	18.39±3.12	18.79±3.76	0.549	0.721	–
Ophthalmological parameters							
Spherical equivalent, dioptre	−1.42±0.66	−1.45±0.71	−1.53±0.70	−1.40±0.72	0.378	0.899	–
Axial length, mm	24.04±0.71	24.20±0.70	24.50±0.82	24.48±0.70	**<0.001**	**<0.001**	Grade 0/1<2/3
Corneal radius of curvature, mm	7.77±0.24	7.83±0.24	7.95±0.26	7.97±0.25	**<0.001**	**<0.001**	Grade 0/1<2/3
Axial length/corneal radius of curvature	3.10±0.07	3.10±0.07	3.08±0.06	3.07±0.07	0.059	**0.030**	–
Macular choroidal thickness, μm	234.07±40.13	205.04±33.24	179.28±29.95	154.15±22.70	**<0.001**	**<0.001**	Grade 0/1>2/3
Macular retinal thickness, μm	278.70±11.03	274.80±11.00	271.54±12.40	273.34±7.95	**<0.001**	**0.001**	Grade 0>1/2/3
Subfoveal choroidal thickness, μm	250.83±51.29	216.96±42.24	187.00±38.21	159.12±27.82	**<0.001**	**<0.001**	Grade 0/1>2/3
Foveal retinal thickness, μm	232.56±16.48	230.48±16.87	229.70±15.41	231.48±15.20	0.152	0.653	–

P values < 0.05 were in bold.

*Analysis of variance or χ^2^ test to compare systemic or ophthalmological parameters in groups with different fundus tessellation grades.

†P trend was acquired by trend test.

‡Post-hoc tests among tessellation groups were conducted with Student-Newman-Keuls method.

In the analysis of variance, those with higher fundus tessellation had longer AL (p<0.001), larger CR (p<0.001), thinner ChT (p<0.001) and macular retinal thickness (p<0.001). There was no significant difference in age, sex, height, weight, BMI, SE or foveal retinal thickness.

### AL, CR and fundus tessellation

**Figure 3:**
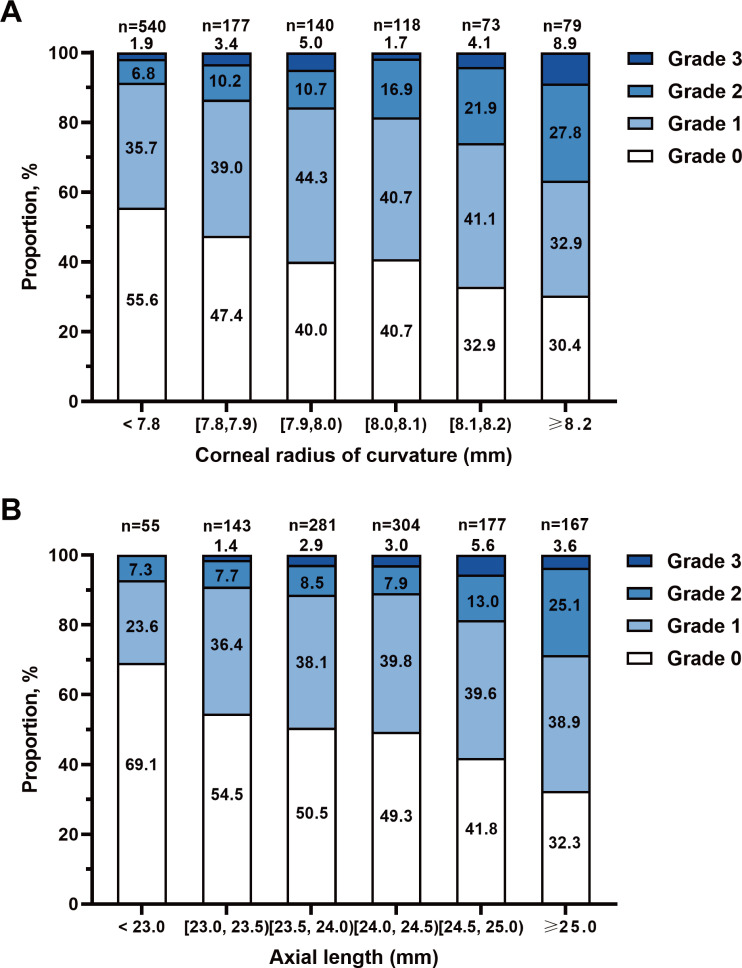
Proportion of fundus tessellation in groups by corneal radius of curvature or axial length. Children with low myopia were grouped according to the corneal radius of curvature (A) or axial length (B), and the proportion of fundus tessellation is shown.

The subjects were grouped according to CR and AL, respectively (figure 3). The proportion of fundus tessellation in participants with AL ≥25.0 mm was significantly higher than in eyes with shorter AL (23.0≤AL<23.5 mm, 45.5%; 23.5≤AL<24.0 mm, 49.5%; 24.0≤AL<24.5 mm, 50.7%; 24.5≤AL<25.0 mm, 58.2%; AL ≥25.0 mm, 67.7%) (p<0.001). The proportion of fundus tessellation gradually increased as the CR became larger (CR <7.8 mm, 44.44%; 7.8≤CR<7.9 mm, 52.5%; 7.9≤CR<8.0 mm, 60.0%; 8.0≤CR<8.1 mm, 59.3%; 8.1≤CR<8.2 mm, 67.1%; CR ≥8.2 mm, 69.6%) (p<0.001).

### Factors influencing macular fundus tessellation grade

**Figure 4:**
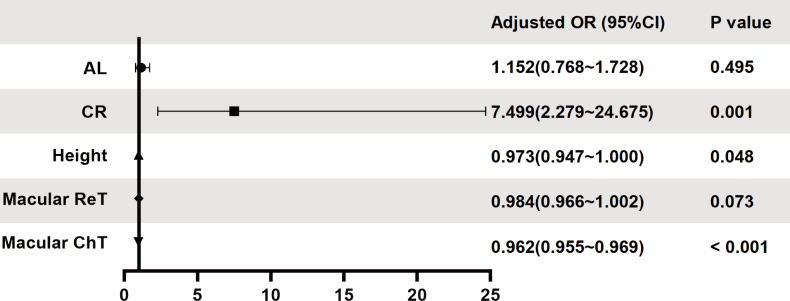
Multivariate regression analysis of fundus tessellation (grades 0–1 vs grades 2–3). A binary logistic regression analysis was conducted to explore factors influencing the degree of macular fundus tessellation (grades 0–1 vs grades 2–3). AL, axial length; ChT, macular choroidal thickness; CR, corneal radius of curvature; ReT, macular retinal thickness.

A binary logistic regression analysis was conducted to explore factors influencing the degree of macular fundus tessellation (grades 0–1 vs grades 2–3). After dropping variables with collinearity, in the analysis of fundus tessellation grades 0–1 vs grades 2–3, a higher degree of macular fundus tessellation (grades 2–3 vs grades 0–1) was independently associated with larger CR (OR, 7.499; 95% CI 2.279 to 24.675, p=0.001), lower height (OR, 0.973; 95% CI 0.947 to 1.000, p=0.048) and thinner macular ChT (OR, 0.962; 95% CI 0.955 to 0.969, p<0.001) (figure 4).

### CR for classifying macular fundus tessellation

**Figure 5:**
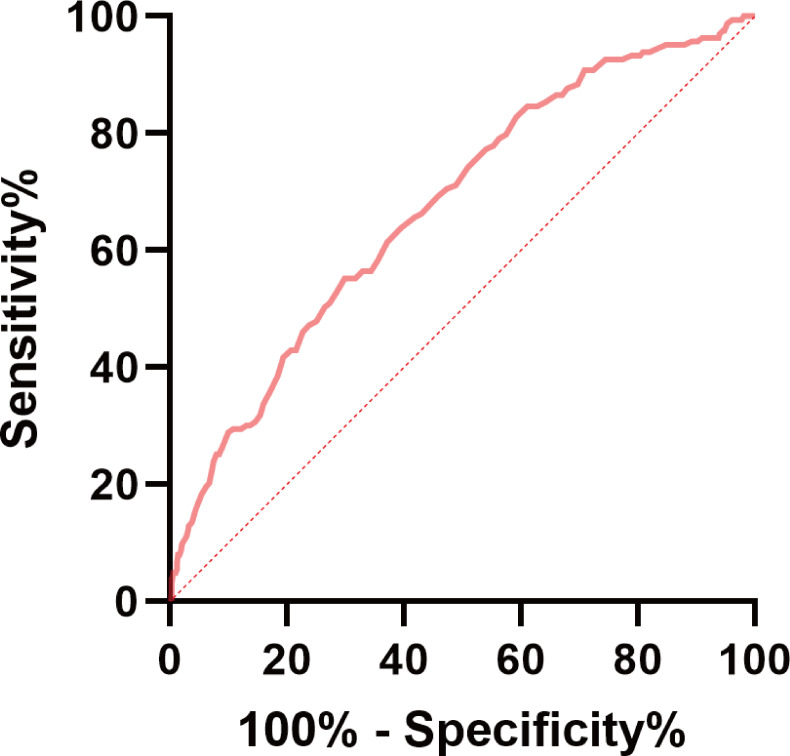
ROC curve of CR for classifying fundus tessellation (grades 0–1 vs grades 2–3). The AUC value was 0.674 (p<0.001). AUC, area under curve; CR, corneal radius of curvature; ROC, receiver operating characteristic.

**Figure 6:**
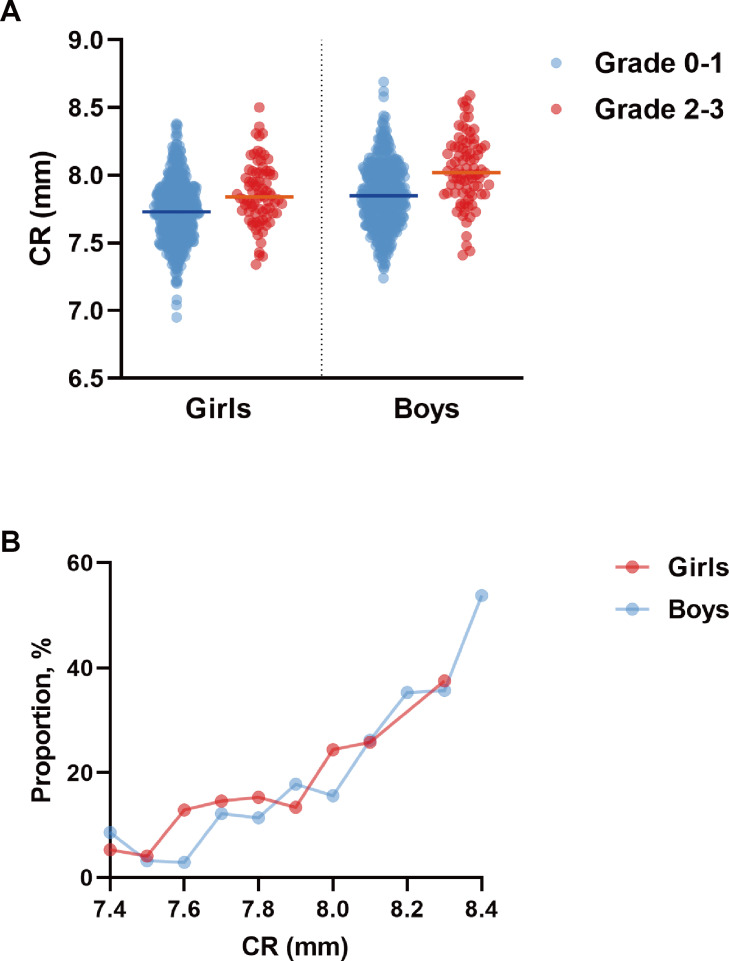
Distribution of CR and proportion of fundus tessellation along with CR. The distribution of CR in total, boys or girls are shown (A). The proportion of fundus tessellation grades 2–3 in each CR group is shown in the line chart (B). CR, corneal radius of curvature.

The receiver operating characteristic analysis for macular fundus tessellation (grades 0–1 vs grades 2–3) with CR was conducted. The area under curve value was 0.674, which was significantly higher than 0.5. The cut-off value of CR for classifying fundus tessellation was 7.912 mm (figure 5). In both girls and boys, the CR of those with fundus tessellation grades 2–3 was much higher than those with grades 0–1 (figure 6A). Along with CR, the proportion of fundus tessellation increased sharply when CR was >7.9 mm (figure 6B).

## Discussion

To our knowledge, this study specifically investigated the prevalence of fundus tessellation in children with low myopia and its associated factors. In this study, we found that 52.4% of children with low myopia had fundus tessellation based on the ETDRS grid-assisting grading method.^17^ In addition, higher fundus tessellation grade was independently associated with larger CR.

The current study found that the prevalence of fundus tessellation in children with low myopia was much higher than expected. More than half of children with low myopia had fundus tessellation and approximately 15% with grades 2–3. In previous studies, Yamashita *et al* 18 reported that, in 1670 Japanese adults aged 40–88 years, 45.4% were with fundus tessellation. Wong *et al* 19 reported that, in 44 adolescents with high myopia aged 12–16 years, the prevalence of fundus tessellation was 54.6%. There seemed to be a similarity in the prevalence of fundus tessellation between the current population of children with low myopia and previous high myopic or elder population. This might be due to the difference in diagnosis or grading method of fundus tessellation. One of our previous studies using the same grading method showed that, in children aged 4–19 years old with high myopia, the prevalence of fundus tessellation was 94.3% and was much higher than that in the current study.^17^

The grading with the ETDRS grid was relatively strict, sensitively reflecting the extent of lesion involving the macula. The onset of myopia in children with low myopia was relatively early, which meant a longer response progression phase and a worse prognosis of myopia; thus, a strict grading method might be more suitable for the current population.

This study verified that a high fundus tessellation grade could reflect a risk of pathological myopia in children with low myopia. The appearance of fundus tessellation was related to a thin macular ChT and a long AL. Excessive AL would result in the stretching of the choroid and retina, which influence both vascular and retinal atrophic changes in the fundus. The findings suggest that children with low myopia with fundus tessellation were potential high-risk candidates for pathological myopia. Accordingly, it has been clarified that the appearance of fundus tessellation suggests a high risk of myopic fundus lesions,^19–22^ and about 20%–30% of patients with fundus tessellation would progress to pathological myopia.^14 15^

Fundus changes in children were not always consistent with the degree of myopia (SE). Unfortunately, the condition of fundus in children with low myopia is usually ignored, as the degree of myopia is slight, and screening for myopic fundus changes mainly focuses on the population with high myopia. The results showed that the cornea was flatter in those with fundus tessellation. The low refractive power of flat cornea would counteract the effect of excessive AL on SE and hence cover up the initiation of fundus changes. In addition, the significant loss of lens power in children might also play a similar role.^23 24^ Thus, the elongation of AL could be concealed and the fundus changes related to excessive AL would be neglected. For children with low myopia with high CR, when the compensatory effect of cornea could not coordinate with AL growth and the progression of myopia begin to accelerate, fundus tessellation may have already existed for a long time.

The current study showed that about 15% (fundus tessellation grades 2–3) of children with low myopia might face an unrecognised high risk of fundus lesions, which represents a very large population in view of the high prevalence of myopia. Therefore, in children with fundus tessellation of grades 2–3, the screening and prevention of pathological myopia may be just as necessary as in those with high myopia. In addition, for the management of myopia in children, refraction parameters such as CR and AL should be taken into consideration and it is crucial to detect excessive AL as early as possible.

The findings provide convenient indicators for the monitoring and management of myopic fundus changes in children with low myopia. Measurement of AL or SS-OCT was not available in many medical institutions and may not be necessary for all children with low myopia. Thus, according to the findings, grading of fundus tessellation could be used to estimate ChT and risk of maculopathy. Besides, a higher degree of fundus tessellation was independently associated with a larger CR, suggesting that children with low myopia with a high CR value were more prone to fundus tessellation. Moreover, the results showed that for those with CR >7.9 mm, there was a substantial increase in the degree of fundus tessellation (grades 2–3). In clinics, the measurement of CR through an autorefractor is relatively regular and therefore it is easy to perform preliminary screening targeting fundus tessellation or long AL according to CR in those with low myopia. Thus, children with low myopia with high CR should regularly take fundus photography.

There were some limitations to the study. First, the population was relatively limited, ranging from 9 to 12 years from Shanghai. Our intended future research will cover subjects from a wider age range and more locations. Second, although the readers were well trained and there was a senior ophthalmologist for quality control, the diagnosis was not guaranteed to be absolutely objective. However, compared with other grading methods, fundus tessellation grading with the ETDRS grid is relatively objective. Third, the current study could not capture the progression of myopic fundus lesions in the population with low myopia; thus, we plan to continue follow-up with the hope of capturing the later progression of fundus tessellation, especially in patients who will go on to develop myopic maculopathy.

In conclusion, more than half of children with low myopia in Shanghai had fundus tessellation by grading with ETDRS grid. There was a tendency towards maculopathy in children with low myopia with large CR, especially those with CR >7.9 mm, who should regularly undergo fundus photography to enable preliminary screening for fundus tessellation. For children with fundus tessellation of grades 2–3, screening and prevention of pathological myopia were just as necessary as in those with high myopia.

## Data Availability

No data are available.
